# Non-Coding RNAs: The “Dark Side Matter” of the CLL Universe

**DOI:** 10.3390/ph14020168

**Published:** 2021-02-21

**Authors:** Marcello Francesco Lingua, Giovanna Carrà, Beatrice Maffeo, Alessandro Morotti

**Affiliations:** 1 Department of Medical Sciences, University of Turin, 10126 Turin, Italy; marcello.lingua@edu.unito.it; 2 Department of Clinical and Biological Sciences, University of Turin, 10043 Orbassano, Italy; beatrice.maffeo@edu.unito.it

**Keywords:** chronic lymphocytic leukemia, Richter’s syndrome, miRNAs, lncRNAs, circRNAs

## Abstract

For many years in the field of onco-hematology much attention has been given to mutations in protein-coding genes or to genetic alterations, including large chromosomal losses or rearrangements. Despite this, biological and clinical needs in this sector remain unmet. Therefore, it is not surprising that recent studies have shifted from coded to non-coded matter. The discovery of non-coding RNAs (ncRNAs) has influenced several aspects related to the treatment of cancer. In particular, in chronic lymphocytic leukemia (CLL) the knowledge of ncRNAs and their contextualization have led to the identification of new biomarkers used to follow the course of the disease, to the anticipation of mechanisms that support resistance and relapse, and to the selection of novel targeted treatment regimens. In this review, we will summarize the main ncRNAs discovered in CLL and the molecular mechanisms by which they are affected and how they influence the development and the progression of the disease.

## 1. Introduction

Chronic lymphocytic leukemia (CLL) accounts for 30% of all cases of leukemia and is the most common leukemia in adults in the Western world [1]. CLL is characterized by the proliferation and accumulation of CD5-positive B-lymphocytes in the peripheral blood, bone marrow, lymph nodes, and spleen [2]. Clinically, CLL occurs in two forms, one indolent, characterized by mutated *IGHV_H_* and low ZAP-70 levels, and one aggressive identified by unmutated *IGHV_H_* and high ZAP-70 levels [2,3]. Furthermore, more than 80% of CLL cases present genomic aberrations [4]. The most frequent chromosomal alterations are deletion of chromosome 13q14.3 (~55%), deletion of chromosome 11q22 (~25%), deletion of chromosome 17p13 (~5–8%), and trisomy of chromosome 12 [4]. In addition, genetic alterations leading to the aberrant expression of several proteins such as NOTCH1, P53, BCL2, and ATM are associated with CLL pathogenesis, drug resistance, and relapse [5]. In particular, *TP53* deletions or knock-out mutations are strong negative prognostic markers in CLL, representing also an evolutionary mechanism of resistance [6]. Whereas, *NOTCH1* mutations in CLL lead to the accumulation of the NOTCH1 intracellular domain (NICD), enhancing BCR signaling response, inducing a more aggressive disease, and favoring Richter’s syndrome transformation [7,8].

However, the biological behavior of CLL as well as the response to treatments does not always correlate with genomic alterations [9]. It has been shown that almost 20% of CLL patients do not present chromosomal aberrations [9]. Other events, such as epigenetic changes and non-coding RNA (ncRNA) alterations, have been reported as possible causes of the outcome of the disease [9]. Over the past decade, several studies have identified deregulated ncRNAs in CLL [10]. Indeed, they are implicated in almost all cellular processes altered in human cancers and also in CLL [10]. Thereby, understanding the dynamics by which this “dark side matter” is involved in the deregulated pathways responsible for CLL development and progression could be beneficial for prognostic and therapeutic purposes. Interestingly, in CLL frequent minimal deleted regions have been associated to MicroRNA (miRNA) losses [11]. Principally, the BCL2 negative regulators miR-15/16 are linked to chromosome 13q14.3 deletion, whereas the alteration of chromosome 11q22 is associated to miR-34b/c cluster loss [12]. Conversely, several other ncRNAs that are deregulated in CLL are not related to genetic abnormalities, but are mainly based on altered epigenetic circuits [10].

In this review, we summarize the different classes of ncRNAs (miRNAs, lncRNAs, circRNAs, and tsRNAs) that are found to be deregulated in CLL for altered genetic and non-genetic mechanisms. We highlight their role as diagnostic and prognostic factors, their association with therapeutic options, and their possible implication as new drugs for the treatment of leukemia. Finally, the significance of non-coding molecules in supporting relapse to treatment and Richter’s syndrome transformation (an aggressive form of lymphoma derived from CLL), has been elucidated.

## 2. MiRNAs

MicroRNAs (miRNAs) are a well-known class of small non-coding RNA molecules with a key role in the regulation of gene expression through their interaction with miRNA responsive elements (MREs) in target mRNAs and the inhibition of their translation [13]. They are frequently deregulated in cancer, where they can act as oncogenes or onco-suppressors and are involved in tumor initiation, progression, and drug resistance [13,14]. The first evidence of the critical role of miRNAs in cancer came from CLL and from patients characterized by chromosome 13q14 deletion [15]. In particular, Croce et al. demonstrated that a minimal deleted region on chromosome 13q observed in about 60% of patients leads to the loss of two miRNAs: miR-15a and miR-16-1 [15,16]. These two miRNAs are direct negative regulators of BCL2 and their down-regulation leads to the increase of BCL2 expression and the consequent inhibition of apoptosis in CLL [16,17]. Another miRNA cluster frequently deleted in CLL is the miR-34b/c cluster on chromosome 11q [12,18]. Moreover, also miR-34a is frequently down-regulated in CLL [19], both miR-34a and miR-34b/c are P53 targets [20]. Consequently, P53 induces apoptosis by partially activation the miR-34-BCL2 axis, and the loss of expression of these miRNAs is associated with resistance against P53-mediated apoptosis [9,20].

On the other hand, several miRNAs deregulated in CLL are not linked to genetic abnormalities but instead are mainly based on altered epigenetic circuits. For example, the miR-17/92 cluster is up-regulated in unmutated *IGHV* CLL [21]. In this case, the increased B-cell receptor (BCR) activation induces MYC expression that drives miR-17/92 expression [9,22]. In turn, this cluster regulates cell cycle progression and proliferation through several targets including CDKN1a, E2F5, IKAROS, PTEN, STATs, TP53INP1, and ZBTB4 [9,22]. The other two miRNAs deregulated in CLL are miR-29 and miR-181, inversely correlating with TCL1 expression [23]. TCL1 is known as an oncogene in aggressive CLL, activating AKT and inhibiting DNA methyltransferases DNMT3A and DNMT3B [24]. Accordingly, miR-29 and miR-181 directly target TCL1 and are characterized by lower expression in aggressive rather than in indolent CLL types [13]. Moreover, lower levels of miR-29 clusters are associated with an increase in the responsiveness of CLL cells to BCR ligation and consequently a shorter survival of CLL patients [25]. Indeed, a recently identified direct target of the miR-29 family is tumor-necrosis factor receptor-associated factor 4 (TRAF4), whereas BCR signaling and the down-modulating miR-29 through cMYC induces TRAF4 up-regulation and CD40-NFKB activation, controlling T-cell interactions in the microenvironment [25].

Furthermore, several other miRNAs have been found to be deregulated in CLL: miR-192 is down-regulated and the direct target CDKN1A/p21 is up-regulated in CLL cells [26]; miR-155 enhances responsiveness to B-cell receptor (BCR) ligation in CLL [27]; miR-338-3p and miR-181b in a cohort of CLL samples inversely correlated with USP7, a de-ubiquitinase inactivating tumor suppressor like P53, PTEN, and FOXO [28].

The early detection of CLL can be useful for diagnostic and prognostic purposes. For this reason, several studies have investigated the role of microRNAs as diagnostic and therapeutic markers. For example, the deregulation of a variety of miRNAs such as miR-15a, miR-21, miR-34a, miR-155, and miR-181b is associated with chromosome 17p deletion [29,30], whereas the expression of other miRNAs such as the miR-15a/miR-16-1 cluster and miR-34b/miR-34c cluster is linked to chromosome 13q and 11q deletion, respectively [13]. Conversely, a different study evaluated miRNA profiles in CLL patients with respect to normal donors [31], showing some miRNAs including miR-34a, miR-155, and miR-342-3p were upregulated and others like miR-103, miR-181a, and miR-181b were downregulated in CLL, defining a miRNA signature as potential early diagnostic biomarker.

Circulating miRNAs can also be detected in the plasma and serum of CLL samples and their number is almost one third higher in CLL plasma samples compared to control plasma [10]. In particular, a series of 14 plasma miRNAs are able to discriminate CLL samples from normal plasma, multiple myeloma, or hairy cell leukemia [32], highlighting a distinct profile that can be used for the early detection of the disease and as a very sensitive biomarker to distinguish CLL from other hematological malignancies. In addition, circulating miR-20a inversely correlates with *ZAP70* status and diagnosis-to-treatment time, with low miRNA levels corresponding with worse prognoses [32]. Accordingly, the circulating level of miR-192 is also significantly reduced in the peripheral blood of CLL patients compared with healthy subjects, suggesting its use as a potential biomarker for the early diagnosis of CLL [26]. MiRNAs in the plasma can also be useful for predicting the response to therapy. For example, before treatment the expression level of miR-155 is significantly higher in patients who failed to achieve a complete remission with respect to those experiencing complete response [33]. Accordingly, in patients with high miR-155 levels before treatment, the overall survival is shorter than in patients with lower levels of miR-155 [33]. Thereby, the use of plasma instead of malignant cells for miRNA profiling offers several advantages: plasma is easily accessible and RNA extraction is technically quick to implement. Moreover, circulating miRNAs could also reflect more accurately the complex interrelationships that facilitate the proliferation of malignant B cells in a precocious stage of the disease, rendering them a potentially powerful tool for very early diagnosis and prognosis.

Besides the free circulation of miRNAs in the plasma, they can also be physiologically encapsulated into exosomes and released into the microenvironment by normal and tumor cells [34]. Interestingly, CLL exosomes have a rich content of miRNAs and a specific miRNA-exosome signature, characterized by the up-regulation of miR-150, miR-155, and miR-29 family members and the down-regulation of miR-223, enabling the significant distinguishing of leukemia patients from healthy donors [35]. Moreover, exosome miR-150 and miR-155 are significantly increased after BCR stimulation, suggesting their possible role as prognostic biomarkers able to discriminate the level of BCR activity in CLL patients [35].

Due to the widespread involvement of miRNAs in cancer and in CLL as tumor-suppressors or oncogenes, they can be used in targeted strategies based on the re-expression of down-regulated onco-suppressor miRNAs or in the silencing of oncogenic miRNAs through siRNA-based strategies [10]. However, miRNAs can also lead to off-target effects due to the fact that each miRNA can regulate multiple genes involved in several signal transduction pathways [10]. In particular, the reintroduction of an miRNA can lead to a “supra-physiological” condition in which the normal targets are saturated or overloaded, redirecting it to secondary targets [36]. Moreover, miRNAs are not stable molecules and rapidly degrade inside the human body. Therefore, they can be modified to increase their stability and they need a delivery vehicle, such as a nanocarrier, that must be able to give an efficient, stable, safe, and tumor-specific in vivo delivery [10,37]. The most promising miRNA that could be used as therapeutic target in CLL is miR-34a, an onco-suppressor deregulated in del17p- and TP53-mutated patients and inversely correlated with prognosis and fludarabine refractoriness [38,39]. Indeed, a miR-34a mimic called MRX34 has been developed thorough the synthesis of double-stranded molecule encapsulated in ionizable liposomes (SMARTICLES) for and efficient and safe delivery [40,41]. Pre-clinical studies in an orthotopic mouse model have shown significant tumor regression and prolonged survival without drug-related side effects [10]. This drug is the first miRNa mimic entering clinical trials with a Phase I study in solid cancer, as well as in patients with hematological malignancies including CLL (ClinicalTrials.gov identifier: NCT01829971). Unfortunately, the trial has been closed early due to serious immune-mediated adverse events that resulted in four patient deaths, although with a manageable toxicity profile in most patients [42]. Thereby, in spite of a strong proof-of-concept for miRNA-based cancer therapy, this class of drugs requires further study in order to achieve safety and effectiveness in treating humans. Finally, few anti-miRNA molecules have been developed as drugs to target oncogenic miRNAs [41]. Among them, Cobomarsen, a locked nucleic acid (LNA)-based oligonucleotide inhibitor of miR-155, is of particular interest [41]. This drug entered Phase II clinical trials for cutaneous T-cell lymphoma (ClinicalTrials.gov identifiers: NCT03837457; NCT03713320), promoting the sustained reduction of lesion burden and improving quality of life [43]. Furthermore, Cobomarsen is currently being used in a Phase I trial in a subset of hematological cancers, including CLL (ClinicalTrials.gov identifier: NCT02580552) [41,43].

## 3. LncRNAs

Protein-coding genes make up 3% of our genome, while 80% is made up of non-coding transcripts with different functions [44]. Long non-coding RNAs (lncRNAs) comprise a large and heterogeneous class of transcripts with a length of approximately 200 bp [45]. In the last decade, various studies have provided evidence to support the role of lncRNAs in the regulation of pathogenetic mechanisms in cancer such as proliferation, angiogenesis, deregulation of the tumor microenvironment, and apoptotic inhibition [46]. In CLL, the deletion of chromosome 13q14 is one of the most common genomic alterations [47]. This deletion, in addition to the impairment of the miR-15/16 locus, is also responsible for the loss of two lncRNAs: DLEU1 and DLEU2 [47]. Although their role in CLL is not entirely clear, recent studies have shown that DLEU1 and DLEU2 may be involved in the transcription of some tumor suppressors responsible for the inhibition of NF-kB activity [48].

Another LncRNA with an important role in CLL is MIAT (myocardial infarction associated transcript), which has been found amplified in malignant mature B cells of CLL patients [49]. MIAT is directly regulated by the transcription factor OCT4, whose expression in CLL is directly correlated with this lncRNA. Furthermore, its expression correlates with greater aggressiveness, promoting progression and contributing to resistance to apoptotic stimuli [49].

The deletion of the region of chromosome 17 in which *TP53* is located is observed in about 10% of newly diagnosed CLL patients, and becomes more frequent in the advanced stages of the disease [50]. In response to DNA damage, P53 regulates two lncRNAs in primary CLL: NEAT1 and LincRNA-p21 (long intergenic non-coding RNA p21) [51,52]. Although the direct implication of these two lncRNAs in CLL has not been investigated, they are involved in TP53-mediated processes. In particular, TP53 induces NEAT1, which is required for the assembly of subnuclear bodies known as paraspeckles [51]. As a consequence, the formation of NEAT1-mediated paraspeckle prevents the accumulation of DNA damage and attenuates the activity of P53 in DNA-damaged cells, influencing cell fate decisions and response to therapy [51].

On the other hand, lincRNA-p21 is inducted by P53 during cell-cycle arrest but does not promote apoptosis or cell senescence. Mechanistically, lincRNA-p21 interacts with MDM2 enhancing the generation of the MDM2-p53 complex and down-modulating P53 target genes [53]. Moreover, lincRNA-p21 is associated with the H3K9 methyltransferase SETDB1 and the DNA methyltransferase DNMT1, sustaining H3K9me3 and CpG methylation at pluripotency gene promoters and linking TP53 to heterochromatin regulation [52].

Likewise, BM742401 is an onco-suppressor lncRNA characterized by the high methylation of its promoter in CLL cell lines and patients compared to control [54]. Functionally, BM742401 over-expression in CLL results in the inhibition of cell proliferation and enhances apoptosis through a Caspase-9-dependent pathway, confirming also the tumor suppressing role of BM742401 in CLL [54].

Finally, Ronchetti et al. studied lncRNA expression profiles in CLL patients with respect to normal B-cell populations isolated from tonsils and peripheral blood [55]. The study revealed 24 potentially clinically relevant lncRNAs significantly deregulated in CLL. Among these, lnc-TOMM7-1, located on chromosome 7p in antisense to the interleukin-6 (IL-6) gene and able to regulate its transcription, promotes proliferation and is involved in the differentiation of B cells [55]. More importantly, the 2-lncRNA independent risk model, based on lnc-IRF2-3 and lnc-KIAA1755-4 expression, is able to stratify early-stage CLL patients into three different prognostic groups [55].

Although many challenges remain to be addressed, in the future oncogenic lncRNAs could be therapeutically targeted by multiple approaches, such as post-transcriptional silencing, genome editing, or by the steric inhibition of RNA-protein interactions and secondary structure formations [56]. For example, a recent study proposed an innovative RNA-based strategy to interfere with the oncogenic lncRNA HOTAIR in tumor cells, preventing HOTAIR/EZH2-mediated epithelial-to-mesenchymal transitions [57]. This strategy is based on the use of an HOTAIR mutant form, named HOTAIR-sbid, which is depleted of the EZH2-binding domain and acts as a dominant negative of the endogenous HOTAIR [57]. Conversely, onco-suppressor lncRNAs can be artificially designed and delivered into leukemia cells to bind oncogenic microRNAs and compete with the corresponding mRNA target genes [58].

## 4. CircRNAs

Circular RNAs (circRNAs) are a class of non-coding RNAs with closed ends covalently originating from splicing processes known as back-splicing [59]. Their circular structure makes them much more stable than normal ncRNAs and highly conserved between species [60]. Located predominantly in the cytoplasm, their expression and distribution are often altered in cancer [60]. CircRNAs affect various cellular processes through the induction of mRNA transcription or through acting as sponges (ceRNAs) for some miRNAs [61]. Although their role in the hematopoietic compartment is increasingly being recognized, circRNAs’ expression and function related to CLL remain largely unclear [59]. Interestingly, the majority of circRNAs produced by plasma cells come from Immunoglobulin (Ig) genes [62], creating speculation on their possible differential functions in CLL related to *IGHV_H_* mutational status.

A connection between circRNAs and CLL was initially indicated in a case report on a rare but recurrent t(8;13)(q24;q14) translocation causing the loss of PTV1 locus and the up-regulation of cMYC [63]. Fifty-two ncRNA variants are encoded by PTV1, including 6 microRNAs, with 26 linear and 26 circular isoforms [64]. Both PTV1 and circPTV1 are involved in hematological malignancies and responsible for the negative feedback regulation of cMYC [64], suggesting a potential role of these non-coding RNAs in CLL.

Recently, three major circRNAs have been implicated in CLL pathogenesis. The first identified is circCBFB [65]. CircCBFB is over-expressed in CLL cells and its silencing induces a proliferative block and an increase of apoptosis. Mechanistically, circCBFB acts as a sponge for miR-607, which in turn targets the FDZ3 factor. Specifically, the segregation and inhibition of miR-607 by circCBFB induces FDZ3 over-expression and the consequent activation of the Wnt/β-Catenin signaling pathway, promoting CLL progression [65].

A very recent study identified six circRNAs over-expressed in the plasma of CLL patients compared to those obtained from healthy donors: circ-RPL13A, circ-NACA, circ-ZNF680, circ-RPS10-NUDT3, circ-ZNF514, and circ-RPL15 [66]. Among these, circ-RPL15 is over-expressed especially in patients with unmutated IGHV_H_, indicating its relevance in this context. Using the circBank database, the authors showed that circ-RPL15 may interact with various miRNAs including miR-146b-3p. Specifically, circ-RPL15 sponges miR-146b-3p, which is in turn able to target RAF1. Therefore, circ-RPL15 induces an up-regulation of RAF1 in high-risk patients activating the RAS/RAF1/MEK/ERK pathway [66].

Finally, circ-0132266 has been identified among the circulars that act as onco-suppressors in CLL [67]. This circRNA is down-modulated in CLL patients and its expression is inversely correlated with miR-337-3p, for which it can act as a sponge. Meanwhile, a direct target of miR-337-3p is the pro-myelocytic leukemia protein (PML). Indeed, RNA interfering (RNAi) of circ_0132266 induces an increase in PML, while the inhibition of miR-337-3p represses PML expression, defining a regulatory mechanism in CLL progression [67].

## 5. TsRNAs

TRNA-derived small RNAs (tsRNAs) are a new class of small RNAs derived from transfer RNAs during tRNA processing [68]. In particular, precursor tRNAs (pre-tRNAs) are transcribed by RNA polymerase III and then cleaved by RNase P and ribonuclease Z (RNase Z) so that the correctly processed tRNAs leave the nucleus through a nuclear receptor-mediated export process. The 3′-end cut of pre-tRNAs by endonuclease RNase Z produces these tsRNA molecules starting at the 3′-ends of tRNAs and ending at the stop signal for RNA polymerase III [68,69]. tsRNAs are not merely tRNA degradation debris but have been recognized to play regulatory roles in many specific physiological and pathological processes [68].

In CLL, a non-coding cluster originally called miR-4521/3676 is actually associated with tRNA sequences and represents two tsRNAs: ts-3676 and ts-4521 [70]. Interestingly, ts-3676 is a powerful regulator of TCL1 expression targeting three consecutive 28-bp repeats within the 3′ UTR of TCL1, and the cluster is co-deleted with *TP53* in del17p CLL patients [71]. Moreover, tsRNAs are physically similar to piRNAs as they have a single-strand short RNA without a secondary structure [69]. PiRNAs bind to piwi proteins to form piRNA/piwi complexes that are involved in transcriptional and post-transcriptional gene silencing, binding genomic DNA or mRNA targets [72]. Ts-3676 and ts-4521 can bind both PiwiL2 and Ago1/Ago2 complexes, demonstrating their dual functions as microRNAs as well as piRNAs [70]. Furthermore, a tsRNA expression signature defines the other two tsRNAs, ts-46 and ts-47, which are both strongly down-regulated in CLL [73].

## 6. Non-Coding RNAs in CLL Resistance and Richter’s Syndrome Transformation

Although in many cases CLL is an indolent disease with a long-lasting clinical course, many patients show resistance to therapy and relapse after treatment either with classical chemotherapy-based therapy or new targeted therapies [5,74]. Therapeutic agents targeting the BCR signaling (like Ibrutinib, Idelalisib, and Duvelisib) or BCL2 inhibitors (Venetoclax) have dramatically changed the treatment options for CLL in the last few years [5]. However, drug-resistant clones and clinical relapse have been observed also with these new drugs [75].

Therapy refraction and relapse might be explained by the abnormal expression of non-coding RNAs, particularly in cases where there is not a clear genetic mechanism of resistance. For example, miR-34a is down-modulated not only in patients with a poor prognosis associated with 17p deletion or *TP53* mutation, but also in fludarabine refractory CLLs [39]. Accordingly, miR-181 family expression is inversely correlated with chemotherapy resistance, and miR-181b expression levels significantly predict treatment-free survival [29]. Conversely, miR-155 and miR-21 are over-expressed in patients with poor prognosis and are able to differentiate fludarabine refractory from sensitive CLL samples [10,29,76].

Furthermore, as described previously, the loss of miR-15/16 is an initial event in CLL pathogenesis causes the overexpression of BCL2, whereas the recently developed BCL2 inhibitor Venetoclax shows a 80% response rate also in poor prognostic patients showing 17p deletions [11,69]. A newly identified target of miR-15/16 is ROR1, a WNT5A receptor that is not expressed in normal adult tissues but is highly expressed in CLL lacking this miRNA cluster [77]. Consequently, the combination therapy based on Venetoclax and anti-ROR1 antibodies (Cirmtuzumab) has a synergistic effect on CLL cell viability [77].

Although the role of miRNAs has been investigated in the evolution of drug resistance and refractoriness in CLL, little is known about the implication of other non-coding RNAs such as lncRNAs and circRNAs. Notwithstanding, a recent paper generated a lncRNA transcriptional profile in early-stage CLLs significantly associated with progression-free survival [55]. In particular, two lncRNAs, lnc-IRF2-3 and lnc-KIAA1755-4, define a risk model that is able to stratify CLL patients into three different prognostic groups, in which the high-risk group is characterized by the concomitant high expression of both lncRNAs [55], suggesting their possible implication in relapse.

Richter’s syndrome (RS) is the transformation of CLL into an aggressive lymphoma, most commonly a diffuse large B-cell lymphoma (DLBCL) [8,78]. This highly aggressive form of lymphoma occurs in approximately 2–10% of CLL patients, is often refractory to treatment, and is characterized by a poor outcome of 8–14 months [78]. Approximately 80% of cases are clonally related to the underlying CLL, whereas the remaining 20% of patients have a clonally unrelated DLBCL with a better prognosis [78].

Few non-coding RNAs and in particular miRNAs have been associated to Richter’s transformation. In a recent study evaluating differential gene expression in two CLL patients at the time of their initial CLL diagnosis and after RS detection, miR-19b represents the non-coding RNA most significantly up-regulated in RS [34]. MiR-19b stimulates the proliferation of CLL cells in vitro and is able to down-modulate P53, mirroring an RS-like biological state. Another study shows that CLL patients that are predicted to develop RS (49 patients) have either an increase of miR-125a-5p expression (~20-fold) or a decrease of miR-34a-5p expression (~21-fold) compared with CLL patients that are not predicted to develop RS (59 patients) [79]. Therefore, a high expression of miR-125a-5p or a low expression of miR-34a-5p can predict ~50% of RS with a false positive rate of ~9%. Finally, the expression signature of miR-21, miR-146b, miR-181b (up-regulated), and miR-150 (down-regulated) has also been associated to Richter’s syndrome transformation [80]. Notably, among them miR-21 is able to increase proliferation and colony formation in CLL and DLBCL cell line models

Despite these results, the biological and functional significances of miRNAs and non-coding RNAs in triggering the evolution of RS with respect to deregulated expression being a mere consequence of the lymphoma transformation, have to be elucidated.

## 7. Conclusions

**Figure 1:**
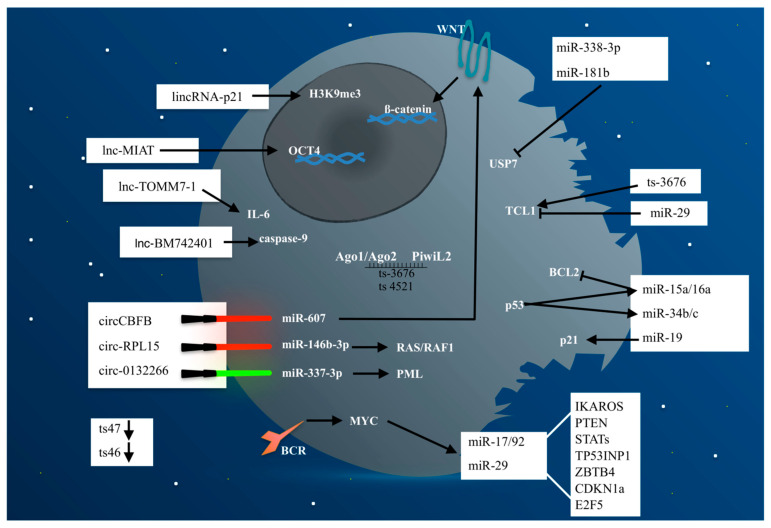
Non-coding RNAs represent the “dark side matter” in CLL pathogenesis. Indeed, although often underestimated they balance the levels of oncogene and onco-suppressor proteins responsible of CLL development and progression. In Figure 1, the CLL cell is symbolized as a “black death” in which ncRNAs (miRNAs, lncRNAs, circRNAs, and tsRNAs) represent the “dark side matter” as opposed to the coding part (“non-dark matter”), both involved in the deregulated pathways responsible for CLL development and progression. The red swords represent a silencing mechanism, whereas green swords represent an inducing mechanism. Arrows indicate activation, whereas bar-headed lines indicate inhibition.

The role of non-coding RNAs, especially in the field of onco-hematology, has represented for years the “dark side matter” of this sector (Figure 1). The identification of altered genes, rather than chromosomal losses or rearrangements, has often marginalized the study of non-coding alterations in CLL, preventing the depiction of their important role in this disease. However, the identification of deletions specifically involving non-coding molecules, in particular miRNAs, allowed us to begin to understand their key function in CLL progression and treatment. Indeed, non-coding RNAs could have a considerable part in the development of new drugs for treatment or could be used for the screening and prevention of CLL evolution. Potentially useful in clinical practice, oncogenic ncRNAs could be directly targeted through interfering RNAs or small molecules that would ensure greater safety for patients. Conversely, stable and safe mimics of onco-suppressor ncRNAs could be delivered to leukemia cells for therapeutic purposes. Of course, despite their undisputed role in the context of CLL, there are many challenges with respect to the coding component that limit the use of non-coding RNAs in the clinical field. However, overall the data obtained so far indicate a high exploitation of ncRNAs in CLL.

## Data Availability

No new data were created or analyzed in this study. Data sharing is not applicable to this article.
